# Wound healing: Natural history and risk factors for delay in Australian patients treated with antibiotics for *Mycobacterium ulcerans* disease

**DOI:** 10.1371/journal.pntd.0006357

**Published:** 2018-03-19

**Authors:** Daniel P. O’Brien, N. Deborah Friedman, Anthony McDonald, Peter Callan, Andrew Hughes, Aaron Walton, Eugene Athan

**Affiliations:** 1 Department of Infectious Diseases, Barwon Health, Geelong, Australia; 2 Department of Medicine and Infectious Diseases, Royal Melbourne Hospital, University of Melbourne, Melbourne, Australia; 3 Manson Unit, Médecins Sans Frontières, London, United Kingdom; 4 Department of Plastic Surgery, Barwon Health, Geelong, Australia; Swiss Tropical and Public Health Institute, SWITZERLAND

## Abstract

**Background:**

Healing times following treatment with antibiotics, and factors that influence healing, have not been reported in Australian patients with *Mycobacterium ulcerans*.

**Methodology/Principal findings:**

Healing times were determined for all *M*. *ulcerans* cases treated by a single physician with antibiotics at Barwon Health, Victoria, from 1/1/13-31/12/16. Lesions were categorised by induration size: category A ≤ 400mm^2^, Category B 401-1600mm^2^ and Category C ≥1601mm^2^. A logistic regression analysis was performed to determine risk factors for prolonged wound healing (>150 days from antibiotic commencement).

163 patients were included; 92 (56.4%) were male and median age was 58 years (IQR 39–73 years). Baseline lesion size [available in 145 (89.0%) patients] was categorised as A in 46 (31.7%), B in 67 (46.2%) and C in 32 (22.1%) patients. Fifty (30.7%) patients had surgery. In those treated with antibiotics alone, 83.0% experienced a reduction in induration size after 2 weeks, then 70.9% experienced an increase in induration size from the lowest point, and 71.7% experienced an increase in ulceration size. A linear relationship existed between the time induration resolved and wound healing began.

Median time to heal was 91 days (IQR 70–148 days) for category A lesions; significantly shorter than for category B lesions (128 days, IQR 91–181 days, p = 0.05) and category C lesions (169 days, IQR 159–214 days, p<0.001). Fifty-seven (35.0%) patients experienced a paradoxical reaction. Of those treated with antibiotics alone, lesions experiencing a paradoxical reaction had longer healing times [median time to heal 177 days (IQR 154–224 days) compared to 107 days (IQR 79–153 days), p<0.001]. On multivariable logistic regression, lesion size at baseline (p<0.001) and paradoxical reactions (p<0.001) were independently associated with prolonged healing times.

For category A and B lesions, healing time was significantly shorter with antibiotics plus excision and direct closure compared with antibiotics alone [Category A lesions median 55 days (IQR 21–63 days) compared with 91 days (IQR 70–148 days), p<0.001; Category B lesions median 74 days (IQR 21–121 days) compared to 128 days (IQR 97–181 days), p<0.001].

**Conclusions:**

In Australian patients treated with antibiotics *M*. *ulcerans* lesions usually initially improve, then clinically deteriorate with increased induration and ulceration, before healing after the inflammation associated with lesions resolves. The time to complete healing of lesions is generally long, and is further prolonged in those with larger initial lesion size or who develop paradoxical reactions. For small lesions (<4cm^2^), excisional surgery may reduce healing times.

## Author summary

In our study we have described for the first time in a large cohort of Australian patients the natural history of wound healing of *Mycobacterium ulcerans* lesions following treatment with antibiotics. We have shown that the lesions usually initially clinically improve with a reduction in induration size, and then deteriorate with increased induration and ulceration, prior to healing after the inflammation associated with lesions resolves. The time to complete healing of lesions is generally long (on average 4.5 months), and is further prolonged in those with increasing initial lesion size or in those who develop paradoxical reactions. In some cases excisional surgery of lesions can reduce healing times by 1–2 months. Knowledge of the natural history of wound healing on antibiotics provides important prognostic information for patients and clinicians when managing *Mycobacterium ulcerans* disease. This information is also needed as a baseline to assess the impact of new interventions aimed at improving healing times. Furthermore, knowledge of factors that affect wound healing times may lead to interventions that significantly reduce them, and in turn the quality and effectiveness of *M*. *ulcerans* treatment.

## Introduction

*Mycobacterium ulcerans* (*M*. *ulcerans)* causes necrotising lesions of skin, soft-tissue and occasionally bone that, if left untreated, usually progress and can lead to significant tissue loss, morbidity and long-term deformity.[1, 2] It has been demonstrated that antibiotics are highly effective at curing *M*. *ulcerans* lesions and preventing disease recurrence,[3–7] and oral antibiotics have reduced hospitalisations and the cost of treatment.[8] Despite antibiotics, healing times can be prolonged, lasting up to 12 months after completion of the recommended 8-week antibiotic regimen if skin defects are large.[6] Prolonged wound healing can lead to significant expense and inconvenience as a result of the need for regular dressings and medical reviews, can be disabling, and can lead to time off work or school resulting in both patient and healthcare provider dissatisfaction.

Healing times in Australian patients with *M*. *ulcerans* treated with antibiotics have not been reported. Healing times in African patients have been reported,[6] but significant differences exist between Australian and African patients that may influence wound healing, with African patients being younger, having fewer co-morbidities, and often less access to care including surgical treatment and appropriate wound care.[9] Furthermore, risk factors for prolonged healing times have not been studied. Knowledge of factors that affect wound healing times may lead to interventions that significantly reduce them, and in turn improve the quality and effectiveness of *M*. *ulcerans* treatment. Understanding the natural history of wound healing with antibiotics provides important prognostic information for patients and clinicians when managing this disease. It is also needed as a baseline to assess the impact of new interventions aimed at improving healing times such as new dressing regimens, new medical treatments or modifications to wound debriding techniques such as medical maggot therapy.

The aim of this study was to describe wound healing times in Australian patients with *M*. *ulcerans* disease treated with antibiotics and to determine risk factors for prolonged healing times.

## Methods

All *M*. *ulcerans* cases treated with antibiotics at Barwon Health, Victoria, from 1/1/13-31/12/16 by a single physician (DOB) were assessed for inclusion in the study. A *M*. *ulcerans* case was defined as the presence of a lesion clinically suggestive of *M*. *ulcerans* plus any of [1] a culture of *M*. *ulcerans* from the lesion, [2] a positive PCR from a swab or biopsy of the lesion, or [3] histopathology of an excised lesion showing a necrotic ulcer with the presence of acid-fast bacilli (AFB) consistent with acute *M*. *ulcerans* infection. The initial size of the lesion was determined by measuring with a ruler the diameter of induration of lesions in millimetres and calculating the surface area in millimetres squared. Lesions were catagorised by the size of induration as follows: category A ≤ 400mm^2^, Category B 401-1600mm^2^ and Category C ≥1601mm^2^. These categories were chosen to allow specific study of small lesions (<2cm diameter and 2–4 cm diameter), common in the Australian context, which would not have been possible using World Health Organization categories. Lesion type and WHO category was assigned according to published definitions.[10]

Wound healing was defined as complete re-epithelialisation of the wound. Healing time was determined as the time from commencement of antibiotic treatment until full healing of the lesion. This was determined by clinical assessment and the examination of photographs of the lesion which could be provided by patients representing times between clinical visits. Lesions were measured every 2 weeks whilst on antibiotics and every 4 weeks once antibiotics were completed. Prolonged healing times were defined as time to healing of more than 150 days after antibiotic commencement.

Standard drug dosages for adults included rifampicin 10 mg/kg/day (up to a maximum of 600 mg daily), clarithromycin 7.5 mg/kg twice daily (up to 500 mg twice daily), ciprofloxacin 500 mg twice daily, and moxifloxacin 400 mg once daily. Surgical definitions were as follows: a) curette: this involved a shallow surgical excision using a curette of the macroscopically abnormal skin and subcutaneous tissue under local anaesthetic with the wound left open to heal by secondary intention; b) debridement: this involved the debridement of necrotic or inflamed tissue in the base of wounds and underlying the wound edges; c) excision and direct closure: this involved excision of the lesion with direct closure of the skin defect; and d) excision and split skin graft (SSG): this involved excision of the lesion with direct closure of the skin defect with a SSG. Treatment decisions were not standardized and were made on an individual patient basis involving of a number of factors which included: initial size, site and type of the lesion, patient co-morbidities and preferences, antibiotic contraindications and intolerances, and lesion response to treatment. Wound care was not standardised and was provided either by medical services or self-administered.

Treatment success was defined as complete healing of the lesion without recurrence within 12 months of commencing antibiotic treatment. Paradoxical reactions were defined by the presence of one or both of the following features: a) clinical: an initial improvement on antibiotic treatment in the clinical appearance of a *M*. *ulcerans* lesion followed by a clinically significant deterioration of the lesion or its surrounding tissues in terms of oedema or tissue necrosis as determined by a clinician experienced in the antibiotic treatment of *M*. *Ulcerans* (DOB), or the appearance of a new lesion(s), and b) histopathology: examination of excised tissue from the clinical lesion showing evidence of an intense inflammatory reaction consistent with a paradoxical reaction.[11]

### Data analysis

Data was collected prospectively using Epi-info 6 (CDC, Atlanta) and analysed using STATA 12 (StataCorp, Texas, USA). Kernel density estimation methods were used to plot graphs of whole cohort lesion responses to treatment with respect to induration and ulceration. Lowess smoothing method was used to compare amount of induration and time to when healing begins on a graph. Median values for non-parametric variables were compared using the Wilcoxon rank-sum test. A logistic regression analysis was performed to determine crude odds ratios of the association of variables with prolonged wound healing time. Variables with a strong association on crude analysis (p≤0.20) were then included in a multivariable logistic regression model to determine adjusted odds ratios for the association of variables with prolonged wound healing time. The p-values for assessing the strength of the association of each variable with prolonged wound healing time, controlled for all the other variables in the model, were determined by the likelihood ratio test.

### Ethics

This study was approved by the Barwon Health Human Research and Ethics Committee. All previously gathered human medical data were analysed in a de-identified fashion.

## Results

### Baseline characteristics

One hundred and eighty-five patients were treated with antibiotics by Barwon Health clinicians over the study period. Nineteen were managed by other physicians in BH, and three had a lack of recorded data and were excluded. Thus 163 patients were included in the analysis.

Ninety-two (56.4%) were male and median age was 58 years (IQR 39–73 years). Baseline lesion size prior to antibiotic treatment [available in 145 (89.0%) patients] was categorised as A in 46 (31.7%), B in 67 (46.2%) and C in 32 (22.1%) patients. One hundred and eighteen (72.4%) lesions were catagorised as WHO category 1, 20 (12.3%) as WHO category 2 and 25 (15.3%) as WHO category 3. One hundred and thirty-six (83.4%) lesions were ulcers, 11 (6.8%) nodules, 14 (8.6%) oedematous and 2 (1.2%) plaques. Thirty-nine (23.9%) lesions were on upper limb, 119 (73.0%) lower limb, and 5 (3.1%) on the trunk, head or neck. Fifteen (9.2%) patients had diabetes mellitus and 16 (9.9%) were immune suppressed.

### Treatment

One hundred and thirteen (69.3%) patients had antibiotics alone, and 50 (30.7%) had antibiotics plus surgery [4 (8%) curette, 15 (30%) debridement, 21 (42%) excision and direct closure, and 10 (20%) excision + SSG]. The type of surgical treatment according to baseline size of lesion is shown in Table 3.

The median duration of antibiotic therapy was 56 days (IQR 46–56 days). Initial antibiotic regimens were rifampicin and clarithromycin in 129 (79.1%), rifampicin and ciprofloxacin in 32 (19.0%), and rifampicin and moxifloxacin in 3 (1.8%).

### Treatment outcomes

One hundred and fifty-eight (96.9%) patients experienced treatment success. Two patients died prior to wound healing occurring (one of a cerebrovascular accident and one with haematemesis secondary to oesophageal varices). Three patients, all of whom experienced paradoxical reactions, took longer than 1 year to heal (392, 535 and 576 days), but all were cured of their disease, with none requiring additional courses of antibiotics (1 had debridement surgery). No patients experienced limitation of joint movements after wound healing.

### Behaviour of *M*. *ulcerans* lesions during antibiotic treatment

**Fig 1:**
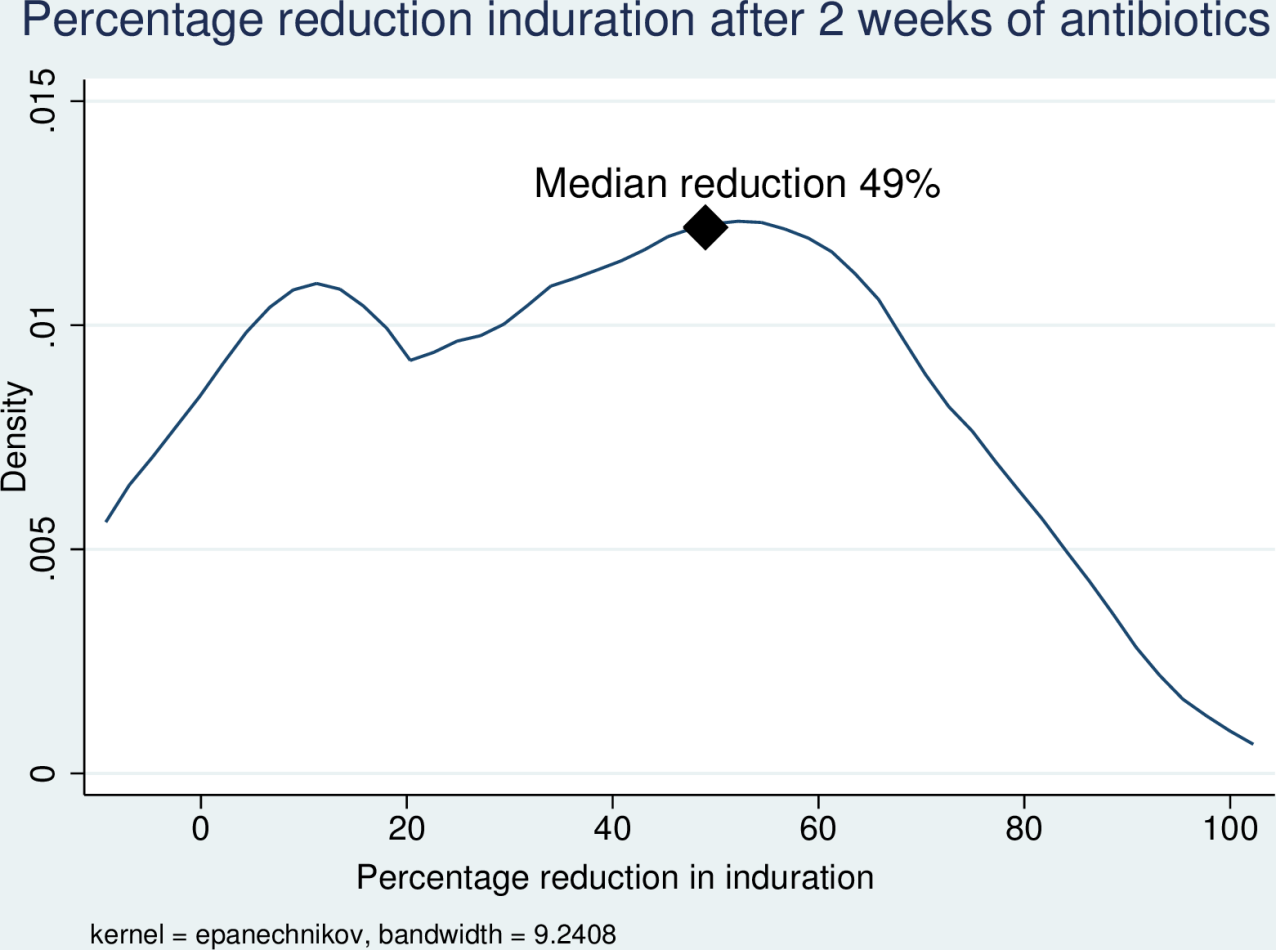
Kernel density graph of percentage decrease in induration size after 2 weeks of antibiotic treatment of *M*. *ulcerans* lesions.

**Fig 2:**
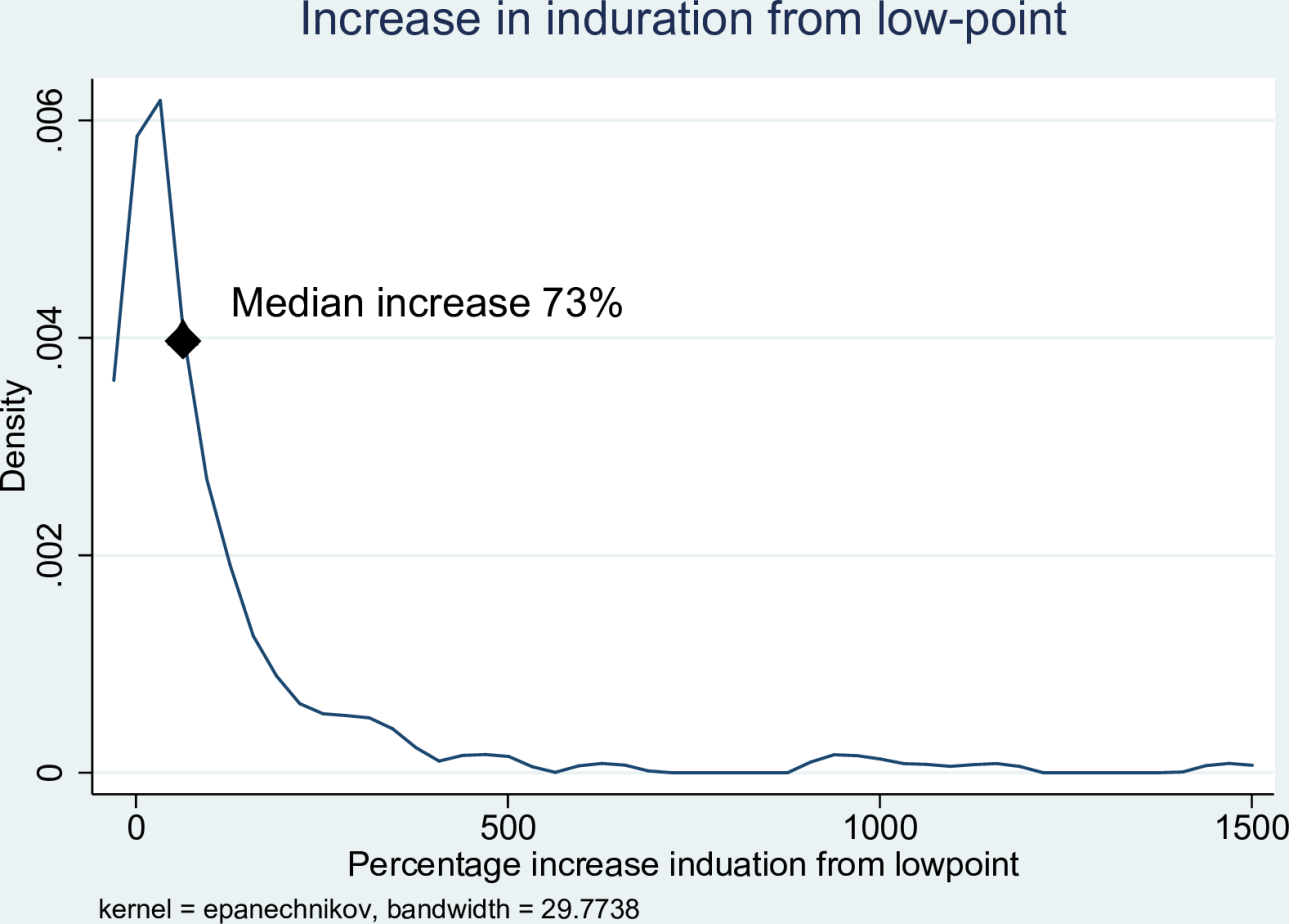
Kernel density graph of percentage increase in induration size from low point following antibiotic treatment of *M*. *ulcerans* lesions.

**Fig 3:**
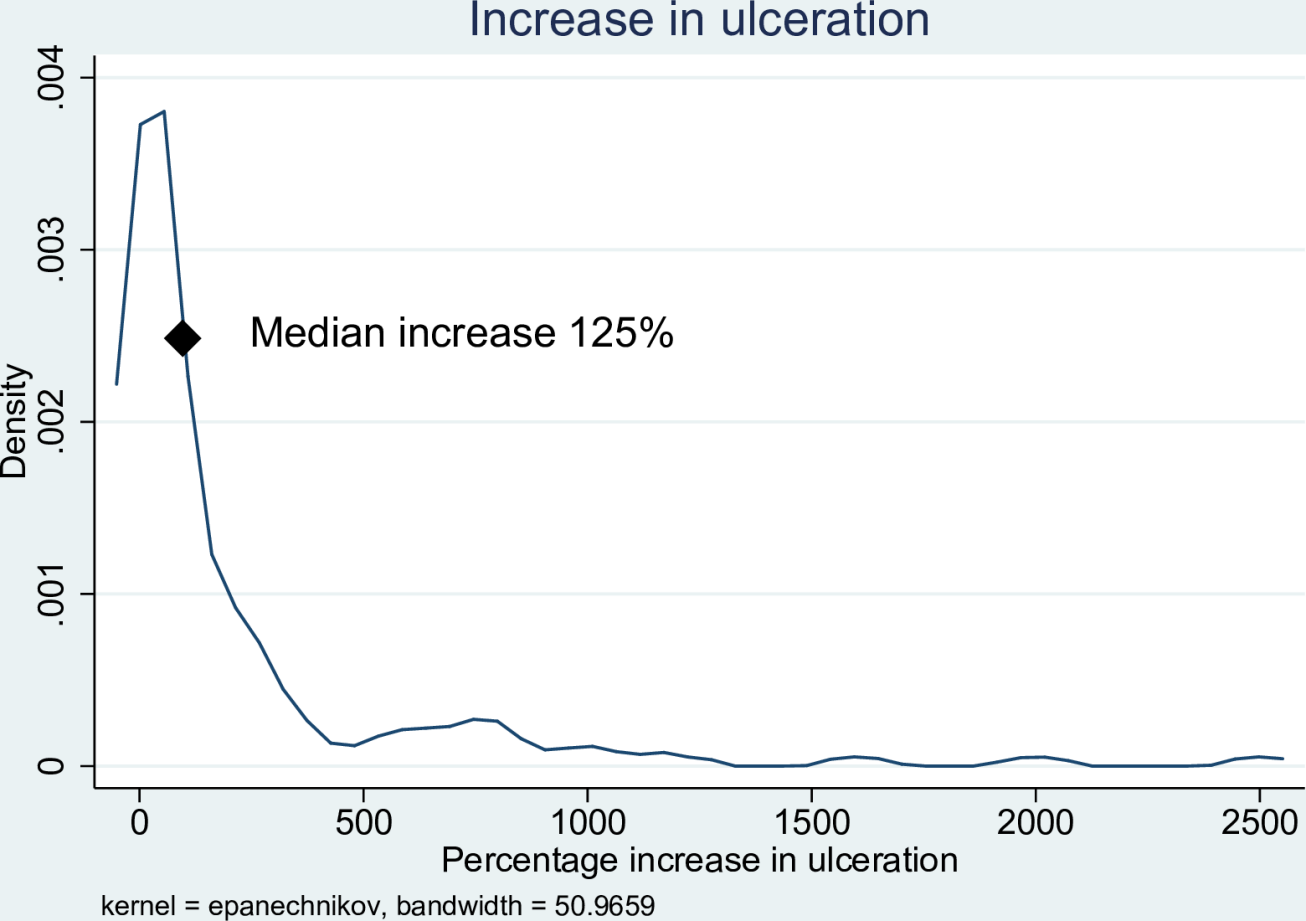
Kernel density graph of percentage increase in ulceration size following antibiotic treatment of *M*. *ulcerans* lesions.

**Fig 4:**
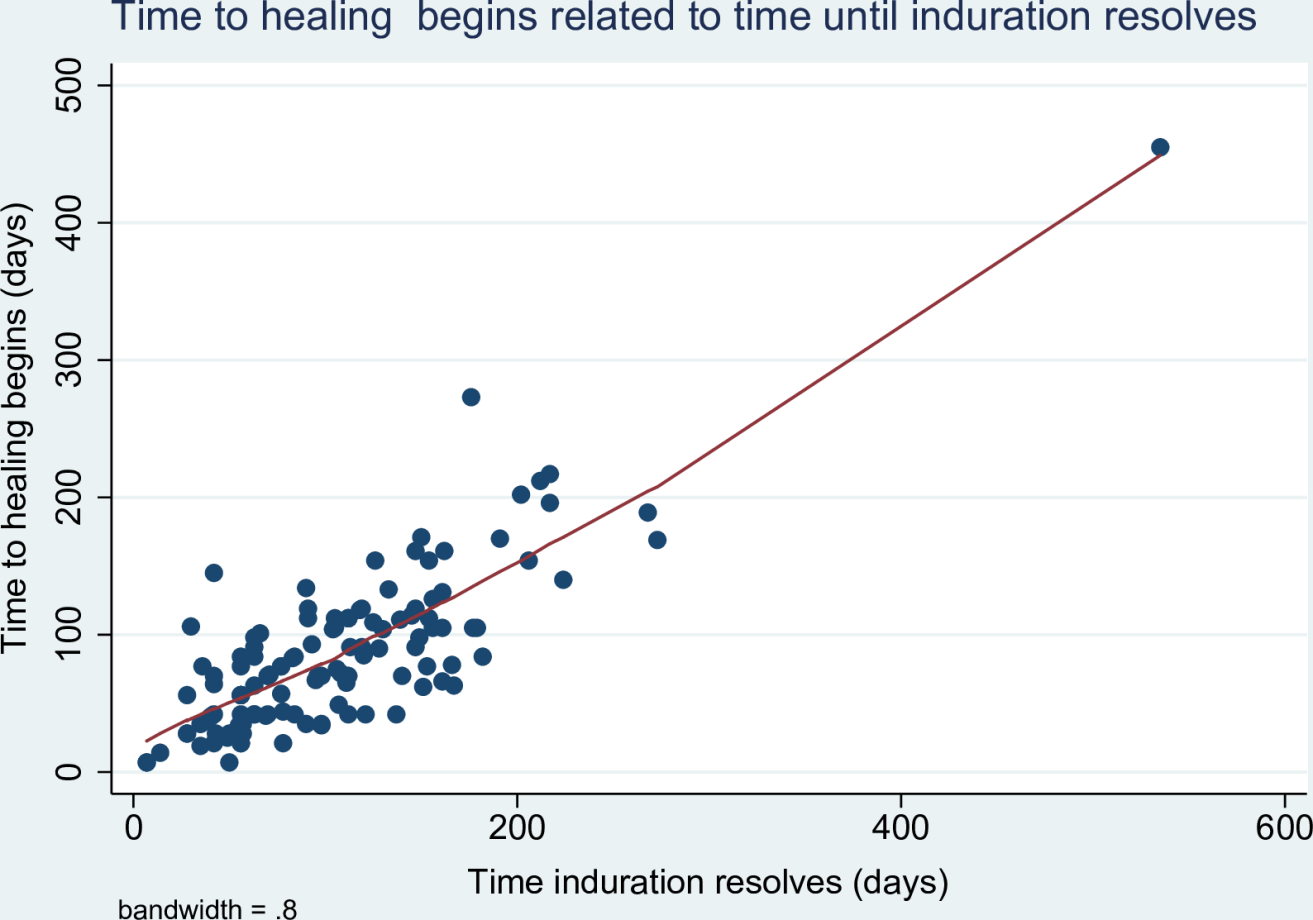
Lowess smoothing graph of time to healing begins related to time until induration resolves for antibiotic treated *M*. *ulcerans* lesions.

In the first two weeks of antibiotic treatment, 98/118 (83.0%) of patients experienced a reduction in the size of induration associated with the lesion, with a median reduction of 49% (IQR 27–63%). (Fig 1) After an initial reduction in the size of induration of the lesion, 95/134 (70.9%) of patients then experienced an increase in the amount of induration from the lowest point [median increase from lowest point 73% (IQR 31–163%)], (Fig 2) with 38/133 (28.6%) of patients developing more induration than at baseline. After antibiotic commencement, 91/127 (71.7%) of patients experienced an increase in the size of the ulceration with a median increased ulceration of 125% (IQR 47–400%). (Fig 3) The induration associated with lesions resolved after a median of 95 days (IQR 56–145 days), and healing of wounds began after a median of 77 days (IQR 42–112 days). There was a linear relationship between the time taken for induration to resolve and the time for wound healing to begin (Fig 4).

### Wound healing

#### Wound healing: Antibiotics alone (Table 1)

The time to wound healing was recorded for 106 (93.8%) patients treated with antibiotics alone: five patients did not have accurate information on time to wound healing, and two died prior to full wound healing, and were not included in further analysis. The median time to heal was 138 days (IQR 91–175 days). Fourty-eight (45.3%) had prolonged healing times, with 3 (2.8%) taking more than 12 months to heal.

**Table 1:** Median healing times of *M*. *ulcerans* lesions treated with antibiotics alone according to baseline characteristics.

Variable	Number of cases	Median healing time, IQR (days)	p-value
Lesion size			
Cat A	31	91 (70–148)	Comparator
Cat B	49	128 (97–181)	**0.01**
Cat C	21	169 (159–214)	**<0.001**
WHO Category			
1	83	119 (84–160)	Comparator
2	14	169 (154–214)	**<0.001**
3	9	175 (152–219)	**0.03**
Gender			
Male	60	143 (93–171)	0.85
Female	46	135 (91–182)	
Age (years)			
0–15	8	113 (88–151)	0.75
16–64	57	119 (84–175)	Comparator
≥65	41	156 (119–182)	**0.03**
Lesion Position			
Upper limb	23	154 (77–202)	Comparator
Lower limb	81	135 (92–175)	0.68
Trunk, head and neck	2	96 (84–108)	0.42
Lesion Type			
Ulcer	99	137 (85–172)	Comparator
Nodule	4	122 (108–174)	0.86
Oedema	2	321 (304–338)	**0.02**
Plaque	1	355 (355–355)	0.10
Diabetes			
Yes	8	154 (140–182)	0.20
No	98	129 (85–175)	
Immune Suppression			
Yes	7	160 (90–168)	0.77
No	97	130 (91–179)	
Paradoxical reaction			
Yes	34	177 (154–224)	**<0.001**
No	72	107 (79–153)	
Drug regimen			
Rifampicin/clarithromycin	86	136 (85–168)	Comparator
Rifampicin/ciprofloxacin	19	153 (11–222)	0.08
Other regimen	1	70 (70–70)	0.24

The median time to heal was 91 days (IQR 70–148 days) for category A lesions, which was significantly shorter than for category B lesions (128 days, IQR 91–181 days, p = 0.05) and category C lesions (169 days, IQR 159–214 days, p<0.001). Median healing times were significantly shorter for WHO category 1 lesions (119 days, IQR 84–160 days) compared with Category 2 (169 days, IQR 154–214 days, p<0.001) or category 3 lesions (175 days, IQR 152–219 days, p<0.01).

Median healing times were significantly longer for patients ≥65 years of age compared to those 15–64 years of age (p = 0.03). Compared to ulcerative lesions, oedematous lesions had significantly longer healing times (p = 0.02). Median healing times were similar between genders, those with and without diabetes or immune suppression, lesion position, and antibiotic regimen.

#### Risk factors for prolonged healing times with antibiotic treatment alone (Table 2)

On crude analysis, lesion size, age, and paradoxical reactions were significantly associated with prolonged wound healing times. Gender, lesion position, lesion type, diabetes and drug regimen were not associated with prolonged healing times. On a multivariable logistic regression, adjusting for age, gender and immune suppression, lesion size at baseline (p<0.001) and paradoxical reactions (p<0.001) were independently associated with prolonged healing times.

**Table 2:** Logistic regression model showing adjusted and unadjusted associations with baseline variables and prolonged healing times.

Variable	Number (%) with prolonged healing	Number (%) without prolonged healing	Crude OR (95% CI)	p-value	Adjusted OR (95% CI)*	p-value
Lesion size						
Cat A	7 (22.6)	24 (77.4)	1	<0.001	1	<0.001
Cat B	21 (42.9)	28 (57.1)	2.6 (0.9,7.1)		2.6 (0.6,10.9)	
Cat C	17 (81.0)	4 (19.1)	14.6 (3.7,57.7)		24.2 (3.8,155.0)	
Gender						
Female	19 (41.3)	27 (58.7)	1	0.47	1	0.40
Male	29 (48.3)	31 (51.7)	1.3 (0.6–2.9)		0.6 (0.2,2.0)	
Age (years)						
0–15	2 (25.0)	6 (75.0)	1	0.01	1	0.21
16–64	20 (35.1)	37 (64.9)	1.6 (0.3–8.8)		0.2 (0.0,1.8)	
≥65	26 (63.4)	15 (36.6)	5.2 (0.93,29.1)		0.6 (0.1–4.4)	
Lesion Position						
Upper limb	13 (56.5)	10 (43.5)	1	0.23	-	-
Lower limb	35 (43.2)	46 (56.8)	0.6 (0.2,1.5)		-	-
Trunk, head and neck	0 (0.0)	2 (100.0)	N/A		-	-
Lesion Type						
Ulcer	44 (44.4)	55 (55.6)	1	0.23	-	-
Nodule	1 (25.0)	3 (75.0)	0.4 (0.0,4.1)		-	-
Oedema	2 (100.0)	0 (0.0)	N/A		-	-
Plaque	1 (100.0)	0 (0.0)	N/A		-	-
Diabetes						
Yes	5 (62.5)	3 (37.5)	2.1 (0.5,9.4)	0.31	-	-
No	43 (43.9)	55 (56.1)	1		-	-
Immune Suppression						
No	41 (42.3)	56 (57.7)	1	0.13	1	0.23
Yes	5 (71.3)	2 (28.6)	3.4 (0.6,18.5)		4.4 (0.4,47.9)	
Paradoxical reaction						
No	19 (26.4)	53 (73.6)	1	<0.001	1	<0.001
Yes	29 (85.3)	5 (14.7)	16.2 (5.5,47.8)		27.4 (6.9,107.9)	
Drug regimen						
Rifampicin/clarithromycin	37 (43.1)	49 (57.0)	1	0.33	-	-
Rifampicin/ciprofloxacin	11 (57.9)	8 (42.1)	1.82 (0.67,4.98)		-	-
Other regimen	0 (0.0)	1 (100.0)	N/A		-	-

* Adjusted for age, gender, size of lesion, immune suppression and paradoxical reaction

#### Wound healing: Antibiotics plus surgery (Table 3)

The overall median time to heal following antibiotics plus surgery was 119 days (IQR 57–159 days). Median healing time was 71 days (IQR 64–114 days) if curette was performed, 137 days (IQR 117–243 days) if debridement was performed, 55 days (IQR 21–113 days) if excision and direct closure was performed, and 152 days (IQR 121–223 days) if excision and SSG was performed.

**Table 3:** Type of surgical treatment according to baseline size of lesion.

Size category	Curette	Debridement	Excision + direct closure	Excision + SSG	Total
A	4	0	9	0	13
B	0	6	6	2	14
C	0	6	0	4	10
Total	4	12	15	6	37

For category A lesions, healing time was similar for those treated with antibiotics plus curette compared with those treated with antibiotics alone (median 71 days with curette compared with 91 days without curette, p = 0.32). However antibiotic treatment duration was shorter for curetted patients [median 20.5 days (IQR 16.5–24.5 days) compared to 56 days (IQR 56–56 days), p<0.01] with no difference in treatment outcomes.

For category A and B lesions, healing time was significantly shorter for those treated with antibiotics plus excision and direct closure compared with those treated with antibiotics alone [Category A lesions median 55 days (IQR 21–63 days) compared with 91 days (IQR 70–148 days), p<0.001, and Category B lesions median 74 days (IQR 21–121 days) compared to 128 days (IQR 97–181 days), p<0.001]. In addition, antibiotic treatment duration was shorter for patients treated with excision and direct closure [median 28 days (IQR 25–42 days) compared to 56 days (IQR 56–56 days), p<0.001] with no difference in treatment outcomes.

There was no significant difference in healing times for category B (p = 0.71) or category C lesions (p = 0.24) treated with antibiotics plus surgical debridement compared with antibiotics alone. However 75% of lesions treated with debridement experienced a paradoxical reaction. Likewise there was no significant difference in healing times for category B (p = 0.48) or category C lesions (p = 0.77) treated with antibiotics plus excision + SSG compared with antibiotics alone.

### Wound healing: Paradoxical reactions

Fifty-seven (35.0%) patients experienced a paradoxical reaction after a median 62 days (IQR 35–87 days) from start of antibiotic treatment. Twelve (21.0%) were diagnosed by clinical and histological criteria and 45 (79.0%) on clinical criteria alone. Of those treated with antibiotics alone, lesions affected by a paradoxical reaction had longer healing times than those not experiencing a paradoxical reaction [median time to heal 177 days (IQR 154–224 days) compared to 107 days (IQR 79–153 days), p<0.001]. This was true for each size category of lesion: Median 165 days (IQR 139–206 days) compared to 84 days (IQR 70–119 days) for Category A lesions (p<0.01), median 178 days (IQR 151–224 days) compared to 106 days (84–128 days) for Category B lesions (p<0.001), and median 191 days (IQR 167–347 days) compared to 168 days (IQR 137–214 days) for Category C lesions (p = 0.06).

## Discussion

We have described the natural history of *M*. *ulcerans* lesions following treatment with antibiotics in Australian patients. We have shown that for the majority of lesions the following changes occur: in the first weeks of treatment a significant reduction occurs in the size of induration surrounding a lesion. Following this, the size of induration once again increases, and can often become larger than at the start. Additionally, despite ongoing antibiotic treatment, the size of the ulceration increases for most patients. Over time, the induration around the ulceration resolves and at that time, but usually not before, the wound begins to heal. Total healing time is long, with the majority of lesions taking more than 4 months to heal, and is longer in patients with larger lesions or in those who experience paradoxical reactions.

In essence, the clinical appearance of the lesion usually gets worse on treatment, and lesions will not have healed or improved greatly by the time the recommended antibiotic treatment (8 weeks) has been completed. Once the induration has settled the wound will begin to heal. This information is very important for clinicians managing *M*. *ulcerans* as it will allow them to manage their own and their patient’s expectations as to how lesions respond to treatment. It will hopefully also prevent them from mistakenly attributing lesion progression as treatment failure and unnecessarily changing, prolonging or recommencing antibiotic treatment or recommending surgery in pursuit of disease cure. It also provides very important baseline data against which interventions aimed at improving wound healing times can be studied in the future.

Interestingly, healing times of wounds after antibiotic treatment without surgery in our Australian patients were similar to those reported from African patients. In a study by Neinhuis et al in Ghana where patients were treated with antibiotic regimens of 8 weeks, the time to complete wound healing was 18 weeks (95% CI 14–22 weeks) for category 1 lesions and 30 weeks (95% CI 26–34 weeks) for category 2 and 3 lesions.[12] This compares to 17 weeks for category 1, 24 weeks for category 2 and 25 weeks for category 3 lesions in our study. In addition, the behaviour of lesions following antibiotic treatment was similar with 78% of lesions experiencing an increased in size of induration from the low point and in 30% of lesions the amount of induration increased past the size at antibiotic commencement (compared with 71% and 29% respectively in our study). In our study we have created a new sub classification of WHO category 1 lesions as category A (400mm^2^ and Category B (401-1600mm^2^) lesions, and we have shown that there are important differences as far as treatment options (eg surgical interventions) and prognostic outcomes (eg healing times) between these subgroups. We feel that in an Australian setting, where the majority of lesions are WHO category 1 at presentation and surgical services are easily accessible, this is a relevant sub classification and propose that this be considered for adoption in other contexts where it is relevant.

We have identified that a major factor contributing to prolonged healing of wounds is the development of paradoxical reactions and this is independent of the size of the lesion at commencement of antibiotic treatment. Therefore, measures to prevent or minimise them would appear important when trying to improve wound healing. We know that paradoxical reactions are more likely to occur in those who are ≥ 60 years of age, have oedematous lesions and have used amikacin in their antibiotic regimen,[13] but little data is available on how we can prevent them or minimise their adverse effects on wound healing. We have reported success in preventing or minimising tissue necrosis associated with paradoxical reactions using corticosteroids but this needs further study to confirm our findings.[14, 15]

Another important factor affecting wound healing times is the size of lesions at commencement of antibiotic treatment. To improve wound healing times there is thus an urgency to diagnose lesions at an earlier stage of disease. Community campaigns aimed at increasing the awareness of *M*. *ulcerans* and the importance of early diagnosis need to be implemented and linked to medical education facilitating clinicians to recognise and diagnose early *M*. *ulcerans* lesions.[16] This includes atypical forms of the disease such as oedematous and nodular lesions.[2]

In our study we have shown that surgical treatment of lesions with excision and direct closure may reduce healing times for category A or B lesions by an average 1–2 months compared to using antibiotics alone. Additionally, we report that surgical curetting of small lesions (Category A) results in similar healing times to those of similar size treated with antibiotics alone. However, based on our published experience of successful outcomes with the combination of surgery and shortened antibiotic treatment durations,[17] in this study we found that both of these above surgical options have the additional advantage of permitting significantly shorter antibiotic courses (an average reduction of half the duration) to be used without affecting treatment success rates, with the potential to reduce antibiotic related treatment toxicity.[18] Therefore this provides clinicians in settings with access to appropriate surgical services an additional treatment option to antibiotics alone for small lesions that may reduce healing times and antibiotic toxicity. Surgical debridement was not associated with reduced healing times, but any beneficial effect may have been confounded by the fact that the majority of cases where it was used (75%) also experienced paradoxical reactions which would have biased the sample towards prolonged healing. Therefore it is possible that if the cohort had been matched for the occurrence of paradoxical reactions there may have been a beneficial effect on wound healing time for those who had surgical debridement. This needs further study.

Although in a small proportion of patients wound healing took longer than 12 months, it is important to note that in all such cases wounds eventually healed without further antibiotic treatment. This suggests there are a proportion of lesions that will have very prolonged healing times, possibly influenced by low-grade paradoxical reactions which were present in all cases, and in these situations significant patience may be needed on the part of clinicians and patients.

Prolonged healing times can lead to significant patient and clinician dissatisfaction. Therefore there is an urgent need to develop new interventions aimed at improving healing times.[19] Such options could include the use of agents that target the mycolactone toxin of *M*. *ulcerans* in order to minimise tissue destruction associated with lesions, improved dressing regimens and wound care techniques such as negative pressure wound therapy,[20] debridement of wounds with medical maggot debridement therapy,[21] more aggressive surgical debridement techniques, and interventions to prevent paradoxical reactions.[15]

The main limitation of this study is its observational nature where treatment interventions were not randomised. As a result, we cannot make firm conclusions regarding the effectiveness of interventions such a surgical treatment on wound healing rates. In addition, there may have been unrecognised confounders that were not controlled for in the multivariable analysis of risk factors for prolonged wound healing. Nevertheless, the associations with lesion size and paradoxical reactions were very strong and thus we feel that it is unlikely that this would have significantly affected these findings.

### Conclusions

We have described the natural history in response to antibiotic treatment of *M*. *ulcerans* lesions in Australian patients, and have shown that lesions usually initially clinically improve, and then deteriorate with increased induration and ulceration, prior to healing after the inflammation associated with the lesions resolves. The time to complete healing of lesions is generally long, and is further prolonged in those with increasing initial size of lesions or who develop paradoxical reactions. For small lesions (<4cm^2^), excisional surgery can reduce healing times.

## Supporting information

S1 Checklist STROBE checklist. (PDF)
